# Electroporation technique for joint pain – Pilot feasibility study on TMD patients

**DOI:** 10.1002/cre2.327

**Published:** 2020-10-14

**Authors:** Gianluca Martino Tartaglia, Andrea Gizdulich, Marco Farronato, Rishi Jay Gupta, Stephen Thaddeus Connelly

**Affiliations:** ^1^ Department of Medicine, Surgery and Dentistry, Fondazione IRCCS Ca' Granda, Ospedale Maggiore Policlinico University of Milan Milano Italy; ^2^ SST Dental Clinic Segrate Italy; ^3^ San Francisco Veterans Affairs Health Care System, Department of Oral & Maxillofacial Surgery University of California, San Francisco San Francisco California USA

**Keywords:** anti‐inflammatory, electroporation, temporomandibular joint, transdermal drug delivery

## Abstract

**Objective(s):**

It is well appreciated that traditional analgesic delivery routes used to treat pain associated with temporomandibular disorder (TMD) often have harmful unintended side effects as a consequence of systemic distribution. Further, localized delivery of analgesic medication via intra‐articular injections involves a different set of issues limiting their clinical viability. As an option, transdermal analgesic delivery provides for prolonged pain relief and flexibility in dose administration, while limiting systemic exposure and minimizing adverse events. Incorporation of a novel electroporation technique may further increase transdermal drug penetration into synovial tissue/fluid and enhance pain reduction. The present feasibility study compares the effectiveness of an electroporation‐enhanced transdermal application of diclofenac sodium to a conventional intra‐articular injection of triamcinolone acetonide suspension (corticosteroids) to treat patients with TMD associated pain.

**Methods:**

Pre‐ and post‐treatment maximal incisal mouth opening (MIO), pain visual analog scale (VAS) and surface electromyography (EMG) of 22 patients treated with electroporation‐enhanced diclofenac and 37 patients treated with corticosteroids injections were collected and analyzed.

**Results:**

In general, patients treated with electroporation exhibited better results in terms of pain improvement (corrected *p*‐value = .01) compared to the standard treatment, but both methods were similarly effective for improvement of MIO (corrected *p*‐value = .71) and improvement of all EMG indices (corrected *p*‐values ≥ .05).

**Conclusion:**

The enhancing effect of electroporation in transdermal delivery of diclofenac sodium was demonstrated by decreased pain, increase MIO and EMG improvement to normal values. Its analgesic and inflammatory results are comparable with standard treatment offered by corticosteroids.

## 1 INTRODUCTION

Pain associated with temporomandibular joint (TMJ) inflammation has a prevalence of approximately 7–10% in the world population and negatively affects the oral health‐related quality of life of the patients (Dahlström & Carlsson, 2010; Iodice, Cimino, Vollaro, Lobbezoo, & Michelotti, 2019; Ouanounou, Goldberg, & Haas, 2017). The overall goal of treatment is patient pain relief, limiting disease progression, and restoring a compromised function. One of the conventional treatment options for painful TMJ intra‐capsular problems is the intra‐articular injection of different anti‐inflammatory drugs, which are useful to improve articular functionality and eliminate the pain.

Steroidal and non‐steroidal anti‐inflammatory drugs (NSAIDs), corticosteroids, sodium hyaluronate or more recently described platelet‐rich growth factor are some of the substances that have been injected in the TMJ in the aim to decrease pain and inflammation (Guilherme et al., 2019; Haigler et al., 2018; Moldez, Camones, Ramos, Padilla, & Enciso, 2018).

TMJ intra‐articular injection, specifically of NSAIDs and steroids, limits systemic exposure and thus offers the advantage of a reduction in incidence of harmful side effects such as to the stomach. However, there are several disadvantages to intra‐articular injections. It is an invasive procedure that is painful and it has a potential for limited efficacy, particularly on subsequent treatments and because of this there is often decreased patient acceptance and compliance. Thus, there is a need for a different drug delivery system (DDS) to overcome the limitations of adverse events, the variable efficacy and patient compliance problems associated with intra‐articular drug delivery.

Nanostructured lipid carriers (NLC) and transdermal drug delivery (TDD) is a promising DDS able to prolong the delivery, decrease the systemic toxicity of liposoluble drugs and increase patient acceptance and compliance. Additionally, NLC/TDD offers controlled and continuous administration of the drugs, which is particularly useful in drugs with short biological half‐lives (Patel, Patel, Parmar, & Kaur, 2011). The specific physical enhancer used in this study is electroporation, which is the application of short (˂1s), high voltage (50–500 V) pulses causing one of three possible flux enhancing mechanisms – electropermeabilization, electrophoresis or electroosmosis (Wang, Thakur, Fan, & Michniak, 2005). Specifically, electropermeabilization uses short high voltage pulses to increase the electrophoretic mobility, molecular diffusivity and alter the electrical conductivity of stratum corneum by the creation of “defects” ‐ temporary aqueous pores in cell membranes (Denet, Vanbever, & Preat, 2004; Sen, Daly, & Hul, 2002). Stratum corneum is normally resistant to drug penetration due to its electric resistance, electric breakdown potential and flexibility of its coefficient of solubility. Given these characteristics, physical enhancers are effective in overcoming this barrier and make TDD a more clinically useful route of analgesic administration (Hadgarft, 2001). Electroporation can be used to deliver molecules of different lipophilicities and molecular weight. The transport of molecules is possible due to enhanced passive diffusion (Denet et al., 2004).

Diclofenac is a nonsteroidal anti‐inflammatory drug belonging to the phenylacetic acid class and is considered the gold standard for improving joint pain related to arthritis (Wagstaff et al., 2016). Trans‐dermal delivery of diclofenac had been previously researched and reported in humans, however, not by means of electroporation. Results of animal models of inflammatory pain using ultrasound and phonophoresis have be equivocal in decreasing central nociceptive sensitivity (Hsieh, 2009). However, clinical studies using phonophoresis found an increased reduction in soft‐tissue pain associated with knee injuries (Sarma, Hanesh, Yahya, & Mohamed, 2009), while other investigations demonstrated that ultrasound enhancement is effective in minor sport‐related injuries (Rosim, Barbieri, Lancas, & Mazzer, 2005) and iontophoresis is effective when used in healthy adults (Riecke et al., 2011). Importantly, Hartmann et al. (2018) demonstrated an electroporation rat model of acute arthritis showing the added value of electroporation in the transdermal delivery of diclofenac, with an analgesic effect comparable to oral administration when evaluating sensitivity, joint swelling, and cytokine concentration of synovial fluid inflammatory enzymes. The authors of that study provided direct evidence of electroporation‐enhanced transdermal delivery of diclofenac sodium on synovial microcirculation by showing decreased rolling and reduced stickiness of activated leukocytes. Taking into consideration the necessary caution that must be exercised in extrapolating animal data to humans, the previously mentioned studies demonstrating that TDD combined with a physical enhancer is superior to TDD alone along with the direct evidence of superior penetration of diclofenac (Hartmann et al., 2018), there is sufficient rationale for comparing electroporation enhanced TDD against a conventional treatment such as an intra‐articular steroid injection (Bjørnland, Gjaerum, & Møystad, 2007) rather than TDD without electroporation.

Therefore, the purpose of this feasibility study is to investigate if TDD combined with electroporation of diclofenac provides comparable results to direct intra‐articular of triamcinolone (corticosteroids) administration in relieving TMD related pain and improving jaw joint function.

## 2 MATERIAL AND METHODS

This study followed the ethical principles of Declaration of Helsinki guideline and all patients were given a thorough explanation regarding the procedures. After answering all their questions, patients signed a written consent form. The protocol was approved by the ethical committees from each of the two units (IRB012019 MQ03AL01 and UCSFIRB 241418).

**FIGURE 1:**
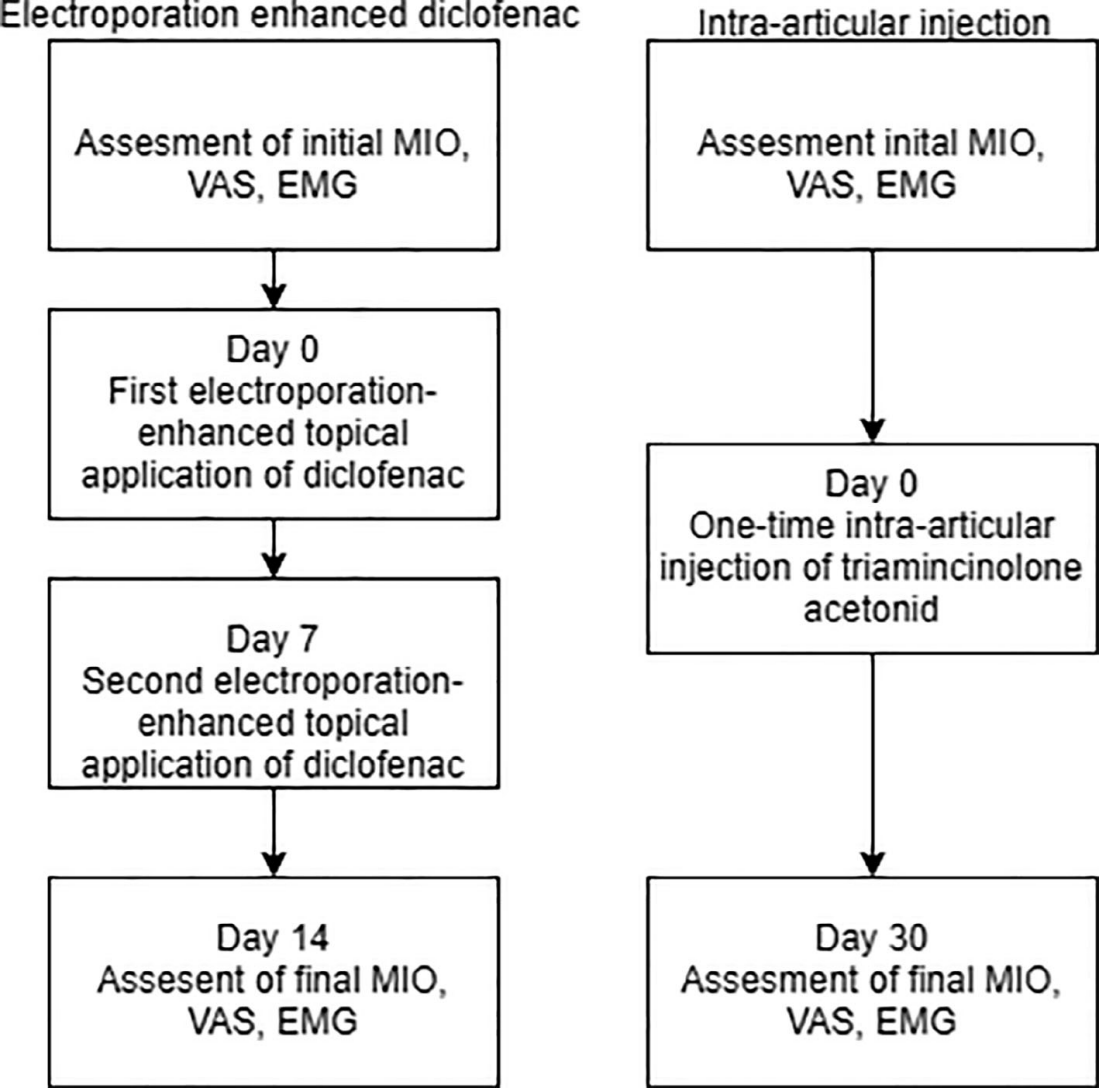
Velvet temporomandibular joint (TMJ)

**FIGURE 2:**
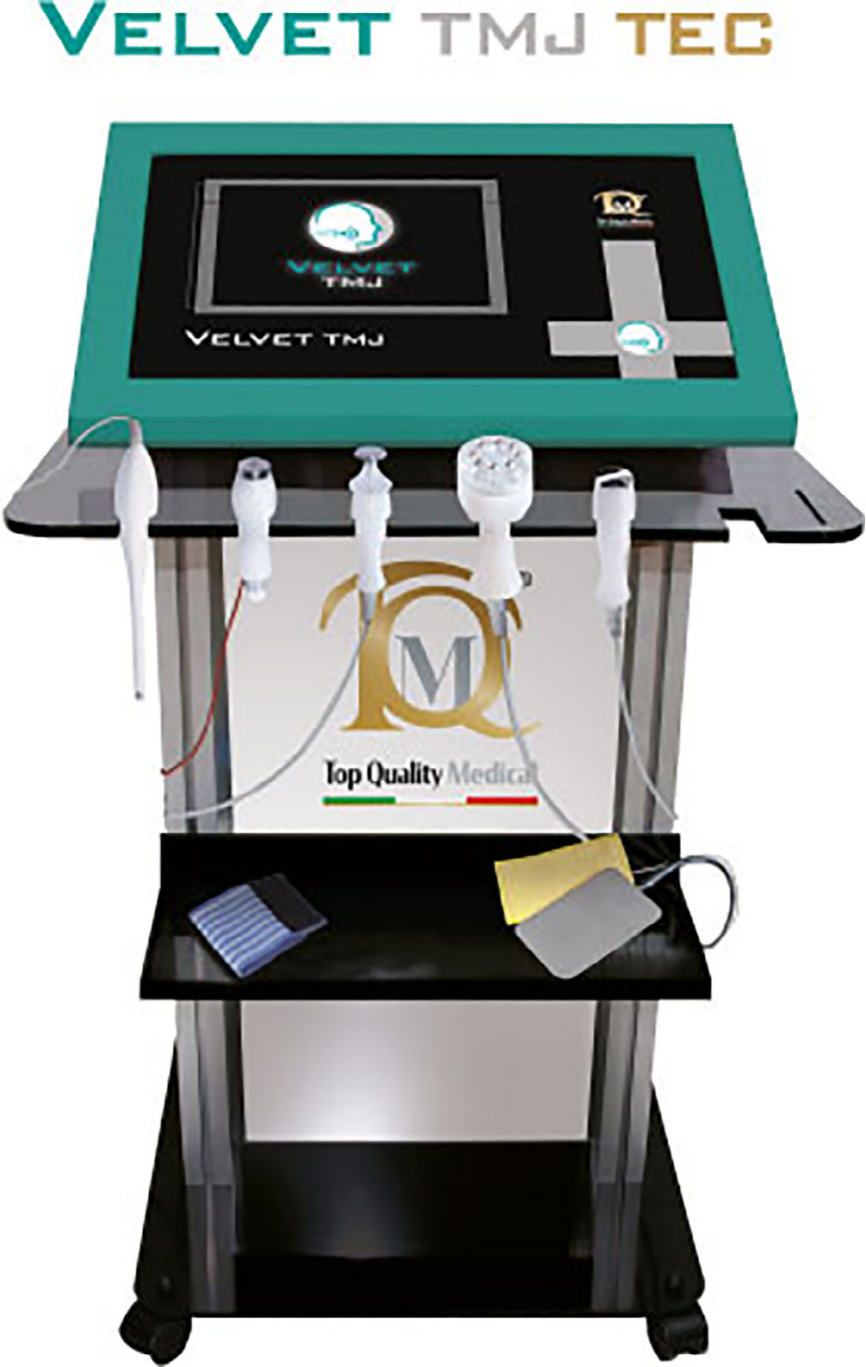
Timeline of the study

The study design is a prospective non‐randomized study comparing two groups of patients who meet the DC/TMD diagnostic criteria of Axis I Arthralgia (Ohrbach, Gonzalez, List, Michelotti, & Schiffman, 2013). Fifty‐three patients per group were planned to be enrolled in the feasibility study in order to reach a higher SP (80%) over period of 6 months. The minimum SP value to be attained for this feasibility study was settled at minimum 50% in set timeframe period. In the TDD/electroporation group, there were a total of 22 patients (21 females and 1 male) with a mean age of 36.5 years (*SD* 13.2 years) who were treated with two electroporation‐enhanced (Velvet TMJ, Top Quality Medical S.r.l., Italy, Figure 1) 15 min topical application of diclofenac 75 mg/3 ml once a week during 14 days (Figure 2). The patients were selected from new patients attending the Department of Gnathology at the SST Dental Clinic, Milano, Italy from January 1, 2019 to the end of June 2019. For the comparator group, there was a total of 37 patients (25 males and 12 females) with a mean age of 53.7 years (*SD* 15.72) who received a one‐time intra‐articular injection of triamcinolone acetonide injectable suspension at the Oral and Maxillofacial Surgery Department at the San Francisco Veterans Health Care System in San Francisco, California, from January 1, 2019 to the end of June 2019 (Figure 2).

Inclusion criteria:

1. Caucasian patients at least or older than18 years of age.
2. Have at least 20 pairs of mastication units on natural or fixed rehabilitated dentition.
3. Not be in active orthodontic treatment in progress (including removable maintenance appliances).

Additional inclusion criteria:

1. A painful joint(s) during mastication (greater than 3 on a visual analog scale (VAS) in which 1 (no pain) and 10 (the greatest possible pain).
2. Limitation during opening (maximal no forced opening with pain <50 mm) or during left and right excursion or protrusion <7 mm.

Exclusion criteria:

1. If patients had received any TMJ treatment in previous the 6 months.
2. If the patients have an existing allergy, sensitivity, cross‐function or contraindications with the experimental drug.

### 2.1 Clinical evaluation

The pre and post treatment assessment of patients was made by a clinician blinded to the actual treatment modality. Patient's clinical history was collected according to the Research Diagnostic Criteria for TMD (Ohrbach et al., 2013). We recorded the pre and post‐treatment status of the following primary outcomes: maximal incisal mouth opening (MIO) defined according to E4_B‐Maximum unassisted opening and pain visual analog scale (VAS). Moreover, patients underwent surface electromyography (EMG) standardized (Ferrario, Tartaglia, Galletta, Grassi, & Sforza, 2006) analysis of their masticatory muscles to supplement the diagnosis, and to monitor the effectiveness of the relevant treatment (secondary outcome) (Ferrario et al., 2006; Tartaglia, Lodetti, Paiva, De Felicio, & Sforza, 2011). EMG activity was recorded using a computerized instrument (Teethan®, BTS S.p.A, Garbagnate Milanese, Italy). EMG signals were recorded and further analysed off‐line.

TMD patients typically exhibit asymmetric contraction of their temporalis anterior muscles, masticatory muscles and unbalanced contractile activity of contralateral masticatory and temporalis muscles (Ferrario et al., 2006), therefore, a set of standardized EMG indices were computed ‐ POC.TA (percent overlapping coefficient temporal anterior muscles standardized bilateral symmetry), POC.MM (percent overlapping coefficient masseter muscles standardized bilateral symmetry), TORS (Torque coefficient ‐ lateral mandible static displacement index), and analyzed (Ferrario et al., 2006). In brief, POC.TA and POC.MM range between 0% and 100%: when two paired muscles contract with perfect symmetry, a POC of 100% is obtained. TORS ranges between 0% (complete presence of lateral displacing force) and 100% (no lateral displacing force) (Ferrario et al., 2006).

### 2.2 Criteria of success

Post‐treatment improvement was analyzed by MIO, VAS and improvement of surface EMG toward normal values measured by two different expert operators 1 week after the treatment by electroporation enhanced diclofenac and 30 days after one‐time intra‐articular injection of triamcinolone acetonide injectable suspension. The time endpoint are settled differently in accordance on electroporation instructions and injection experience where the best outcomes considered were observed on the above mentioned time frame in the two groups. No set point of success, cut‐off values, was defined to observe improvement in MIO, pain and surface EMG values at the end of set timeline (Figure 2).

### 2.3 Statistical analysis

All patient's data were assessed quantitatively in IBM SPSS Statistics 25 software. Nonparametric Wilcoxon signed‐rank test was used to compare the initial and final MIO and VAS scores of each patient, and pre‐ and post‐treatment EMG data. Student *t* test and Fisher exact test were used to compare the age and sex between two groups. Differences between groups in initial and final VAS and MIO scores and EMG values were calculated using by Mann–Whitney *U* test. A two‐sided *p*‐value < .05 was considered statistically significant. Due to multiple analyses on the same sample, Benjamini and Hochberg correction was applied to each calculated *p*‐value. The statistical power (SP) for inter‐group differences of described study design was calculated using GPower software (version 3.1.9.4) and reached 56%. Moreover, the calculated effect size resulted in 0.49 for Mann–Whitney *U* test and 0.24 for Wilcoxon signed‐rank test.

## 3 RESULTS

Twenty‐two and thirty‐seven patients were finally recruited because there was a lack of patients fulfilling inclusion criteria and several patients did not consent. The two groups were composed of Caucasian patients in order to be considered homogenous in terms of ethnicity (the only ethnicity available from the two study locations). The groups significantly differed (*p*‐value < .05) in sex and age distribution from a statistical point of view. Patients treated with electroporation enhanced diclofenac were significantly younger (mean age 36.5 years) compared to patients who received a one‐time intra‐articular injection of triamcinolone acetonide injectable suspension (mean age of 53.7 years). Two groups of patients without significant differences in the initial MIO, VAS and EMG scores were compared (*p*‐value ≥ .05). Table 1 presents descriptive statistics for VAS, MIO, and EMG scores before and after the treatment.

**TABLE 1:** Descriptive statistics of pain visual analog scale (VAS) (in score points), maximal incisal mouth opening (MIO) (mm), and surface electromyography (EMG) values of electroporation and comparator group before and after the treatment

	Corticosteroids injectable suspension (*n* = 37)				Electroporation‐enhanced topical application (*n* = 22)			
	Mean	*SD*	Min	Max	Mean	*SD*	Min	Max
**Pre‐treatment values**								
VAS	6.57	1.69	3.00	10.00	6.41	1.48	3.00	9.00
MIO	35.08	9.17	12.00	50.00	34.45	7.90	22.80	46.90
POC TA	74.0%	18.0%	26.0%	81.0%	74.0%	20.0%	11.0%	90.0%
POC MM	79.0%	13.0%	42.0%	91.0%	74.0%	18.0%	30.0%	90.0%
TORS	82.0%	13.0%	35.0%	93.0%	86.0%	7.0%	66.0%	92.0%
**Post‐treatment values**								
VAS	4.41	1.91	1.00	9.00	1.77	2.25	0.00	7.00
MIO	37.27	7.47	24.00	51.00	40.00	6.56	26.01	47.70
POC TA	84%	7%	60%	89%	85%	3%	79%	89%
POC MM	83%	9%	44%	90%	83%	6%	72%	89%
TORS	88%	5%	71%	92%	90%	2%	86%	92%

After the treatment, significant differences in pain score and MIO were observed in the electroporation group (decrease of pain, corrected *p*‐value = .01 and increase of MIO, corrected *p*‐value = .04). Similar result of pain decrease was observed in the comparator group of patients treated with the conventional care (corticosteroids injections) (corrected *p*‐value = .01), however, the improvement in MIO was not significant (corrected *p*‐value = .10, Table 2).

**TABLE 2:** Results of statistical analysis

	Corticosteroids injectable suspension		Electroporation‐enhanced topical application		Intergroup difference *p*‐value
	Mean	*SD*	Mean	*SD*	
**VAS**					
Pre	6.57	1.69	6.41	1.48	.78
Post	4.41	1.91	1.77	2.25	—
*p*‐value	.01^a^		.01^a^		
Difference in improvement	2.16	1.57	4.64	1.78	.01^a^
**MIO**					
Pre	35.08	9.17	34.45	7.90	.83
Post	37.27	7.47	40.00	6.56	—
*p*‐value	.10^a^		.04^a^		
Difference in improvement	−2.19	6.79	−5.55	6.45	.71^a^
**POC TA**					
Pre	0.74	0.18	0.74	0.20	.61
Post	0.84	0.07	0.85	0.03	—
*p*‐value	.04^a^		.10^a^		
Difference in improvement	−0.10	0.18	−0.11	0.19	.90^a^
**POC MM**					
Pre	0.79	0.13	0.74	0.18	.55
Post	0.83	0.09	0.83	0.06	—
*p*‐value	.10^a^		.11^a^		
Difference in improvement	−0.04	0.10	−0.09	0.18	.30^a^
**TORS**					
Pre	0.82	0.13	0.86	0.07	.12
Post	0.88	0.05	0.90	0.02	—
*p*‐value	.049^a^		.13^a^		
Difference in improvement	−0.06	0.12	−0.03	0.07	.50^a^

^a^ *p*‐value with Benjamini and Hochberg correction applied.

In general, the study showed patients treated with electroporation‐enhanced diclofenac exhibited comparable results in terms of pain and mouth opening improvement as patients treated with the standard treatment. This was confirmed by comparison of an actual improvement in score points (for VAS) and millimeters (for MIO) between two patient groups. The mean improvement in VAS score reached 4.64 VAS points (*SD* 1.78) in electroporation group and 2.16 VAS score points (*SD* 1.58) in the comparator group. The mean improvement in MIO was 5.55 mm (*SD* 6.45) in electroporation group and 2.19 mm (*SD* 6.79) in the comparator group (Table 2).

All patients treated with electroporation enhanced diclofenac delivery reported a decrease in pain and 95% (21/22) of those patients showed also improvement in MIO. One patient showed lower MIO after the treatment. In the comparator group, 86% of patients (32/37) reported a decrease in pain, 10% (4/37) of patients reported the same pain and 1 patient (3.7%) reported increased pain. Similarly, 68% of patients (25/37) showed improvement in the MIO after the treatment, 24% showed (9/37) decreased mouth opening and 8% of patients (3/37) did not show any improvement.

Results of the EMG analysis before and after the treatment with diclofenac enhanced by electroporation are shown in Table 1. The mean values of POC.MM and POC.TA before the treatment were less than 80% in both groups, which indicates asymmetry.22 The mean value of Torque coefficient was in normal range 22 (>80%). In the group of patients treated with electroporation‐enhanced diclofenac, a trend toward improvement was observed in all variables (Table 1); and all indices ranged in normal values after the treatment. However, no significant improvement was observed (corrected *p*‐values > .05). In the comparator group treated with corticosteroids, the improvement was observed in all variables. The statistically significant improvement was detected in a bilateral symmetry coefficient for temporalis muscles (POC TA, corrected *p*‐value = .04) ‐ indices ranged in normal values after the treatment. The significant improvement was also found in Torque coefficient, coefficient (TORS, corrected *p*‐value = .049) however, both pre‐ and post‐ mean values of TORS ranged in normal values over 80%. In terms of improvement, the actual pre‐ and post‐treatment EMG score differences were compared between two patient groups for each observed index. Overall, no significant differences were found in the overall improvements of all indexes (corrected *p*‐values ≥ .05, Table 2).

## 4 DISCUSSION

TDD has gained recent favor as a method to improve pain control and the additional incorporation of transdermal enhancers such as iontophoresis, electrophoresis, ultrasound, needleless injections, and microneedles, or the use of chemical penetration enhancers have proven to provide added benefit. However, investigators remain challenged to invent a delivery system that allows for maximum therapeutic effect, while minimizing adverse effects (Rao, Mahant, Kumar, & Nanda, 2017).

Reversible electroporation, type of TDD, is used for drug or gene delivery and after the end of electric pulses, the transport pores reseal and drug/gene influx ends (Yarmush, Goldberg, Sersa, Kotnik, & Miklavcic, 2014). Above a certain electrical threshold, the temporary pores in cell membranes become permanent (irreversible electroporation) and cause cell death due to the cell's inability to maintain homeostasis (Wagstaff et al., 2016). This previously undesirable effect is currently the main principle behind cell ablation therapy that has demonstrated effectiveness against solid tumors of the liver, pancreas, kidney or prostate cancer (Wagstaff et al., 2016).

To our knowledge, there have been no previous studies of the application of electroporation‐enhanced transdermal delivery of diclofenac to treat any joint disease despite the clear evidence supporting the superior effectiveness of electroporation enhanced transdermal delivery versus transdermal delivery alone. In this paper, we investigate whether or not the analgesic effect produced by electroporation enhanced transdermal delivery of diclofenac is comparable to the standard treatment of an intra‐articular injection of corticosteroids. Drugs belonging to different classes were used due to the fact that it is not possible to enhance corticosteroids by electroporation and the fact that diclofenac is not approved for TMJ injection.

Currently, electroporation is of interest in the field of oncology with the majority of published clinical data (electrochemotherapy or nonthermal cell ablation); cell fusion; gene therapy and DNA vaccination; and for general TDD (Yarmush et al., 2014). However, the interest in the technique and its clinical applications has been expanding to other fields in recent years. For example, a PubMed search of “electroporation” retrieved 246 papers in 1999 and 686 in 2018, with a threefold interval increase (PubMed [Internet], 2019). There are a few publications on anti‐inflammatory drug delivery enhanced by electroporation, however the majority are limited to animal models and use different drugs. Two similar studies applying electroporation enhanced drugs for pain in humans, namely application of sinomenine hydrochloride to the knee (resulted in effective percentage of joint pain relief (VAS) 79.39% ± 4.63%) (Feng et al., 2017) and application of methotrexate injection with and without electroporation on small joints of hand (Jadoul, Bouwstra, & Preat, 1999) can be found.

In the previously mentioned animal study (Hartmann et al., 2018), the added value of electroporation on transdermal delivery of diclofenac in comparison to its simple topical administration was confirmed. Due to the molecular characteristics of diclofenac, its short biological half‐life, and the numerous side effects associated with its oral administration, electroporation seems like a promising technique to overcome all mentioned effects while assuring its sufficient concentration in the synovial fluid to decrease inflammation (Hartmann et al., 2018). As no previous studies of electroporation‐enhanced topical application of diclofenac to any joints exist, we can only hypothesize about possible side effects of its repeated application. General side effects of electroporation had been previously described as sensation or pain due to current applied on the skin; itching, tingling, pricking or muscle contraction. Electroporation can cause changes on molecular levels, such as decrease of skin resistance, increase of hydration; disorganization of the stratum corneum lipid barrier's or increase in blood flow. The only repeated application of electroporation can be currently seen in electrochemotherapy reporting temporary mild side effects described above (Jadoul et al., 1999; Prausnitz, 1996; Sersa, Cemazar, & Rudolf, 2003).

Our presented study has found non‐significant differences in improvement of pain and mouth opening between two groups. This indicates that enhancing the transdermal delivery of diclofenac by electroporation results in comparable effects as the intra‐articular injection of the potent anti‐inflammatory agent, corticosteroids. Moreover, electroporation enhances penetration of diclofenac and one‐per‐week application is sufficient in comparison to usually required multiple‐per day topical application. The effect was demonstrated by decreased pain, increase MIO and EMG improvement compared to normal values of patients. In comparison to corticosteroids injections, electroporation enhanced topical administration of diclofenac is convenient, non‐invasive and painless. Our preliminary results further indicate that electroporation‐enhanced delivery of diclofenac may perform better in decreasing the pain.

In the previous EMG investigations, TMJ disorder patients exhibited alternations in their standardized muscle activities. This was also confirmed in our sample ‐ the mean values of POC.MM and POC.TA indices before the treatment were lower than 80%, which indicates unbalanced standardized activity of masseter and temporalis muscles between left and right (asymmetry) muscle sides. Value 80% is considered as normal function of the masseter and temporal muscles with standardized muscular symmetry (Tartaglia et al., 2011). Unbalanced activity in TMJ patients was previously reported (Landulpho, Silva, Silva, & Vitti, 2004) and it is related and may result from the inhibition of the muscular activity during function to protect TMJ articulation from overloading. In this study, after the treatment by electroporation‐enhanced diclofenac, significant improvement was observed only in the temporalis muscles. Additionally, after the treatment with electroporation‐enhanced diclofenac, averages of all observed EMG variables reached over 80%.

More large‐scale clinical studies are needed to understand and confirm the safety of electroporation in TDD, and its application in the delivery of anti‐inflammatory drugs, or its comparison to other standard treatments as a current study setting was able to detect inter‐group differences of more than 56%. Larger samples are also needed to achieve higher SP – 41, 53, and 73 patients per group to achieve 70, 80, and 90% SP respectively. The cross‐over study design could bring more light into the effectivity of electroporation; however, such a design was not possible for the limits and the uncertainties in the area. We are well aware that these preliminary results and the scientific evidence of this feasibility cohort study do not permit clinical conclusions but, it is the essential first step to move forward and encourage future research with larger numbers of patients (and consequently the economic cost) in larger multi‐centric studies. This fundamental step is essential in order to better establish the procedure limitations as an adjuvant tool in the non‐invasive therapy of our patients.

In the future, all possible variables should be controlled and the influence of age, sex, ethnicity, menstrual cycle or severity of disease should be studied. Side effects of single and repeated application of electroporation to TMJ must be described. Our preliminary data showed the potential of electroporation in the treatment of TMJ diseases and further investigations on this procedure are being planned. Similarly, the study described effects of electroporation‐enhanced diclofenac 1 week after two applications. In the described feasibility study no side effects were observed. Future 6 months recall appointments were planned for patient to evaluate its long‐lasting effects. Future studies of repeated application of electroporation must be also performed to assess safety of such approach and its possible advantages/disadvantages compared to a control group using placebo in order to better define the effect of drug electroporation per se and prognostic time set in TMD arthralgia patients. This is a feasibility study designed to reduce the economical and biological cost and at the same time enlist the highest number of patients looking for a clinical solution in a defined time frame. The lack of a control group, and significant differences in age and sex of patients are the weakness of this protocol and further investigations in this direction are being planned because the feasibility approach was encouraging. This age difference might be caused mainly due to the experimental nature of diclofenac electroporation application, as older patients preferred not to undergo it. On the other hand, this is the first description, to our knowledge, regarding the application of this technique in dentistry and a cautious approach on the study design was planned to reduce the risk of patients' treatment failure.

## 5 CONCLUSION

Acute arthralgia is a well‐identified disease in the taxonomy of TMD problems with very good prognosis with early diagnosis and treatment. TDD is one of the treatment options with two advantages. In the foreground, it is fast and secondly, it is economic, and thus it allows us to help the patients more efficiently. With the limitation of this study, TDD demonstrated to be a useful tool compared to standard intraarticular injection for its non‐invasiveness and minimal discomfort. Further clinical trials are indicated to better define the clinical technique and applications along a broader cohort of TMD patients.

## CONFLICT OF INTEREST

All authors have no conflict of interest.
